# Temperature-Dependent Growth of 36 Inner Nanotubes inside Nickelocene, Cobaltocene and Ferrocene-Filled Single-Walled Carbon Nanotubes

**DOI:** 10.3390/nano11112984

**Published:** 2021-11-06

**Authors:** Marianna V. Kharlamova, Christian Kramberger

**Affiliations:** 1 Institute of Materials Chemistry, Vienna University of Technology, Getreidemarkt 9/BC/2, 1060 Vienna, Austria; 2 Moscow Institute of Physics and Technology, Institutskii Pereulok 9, 141700 Dolgoprudny, Russia; 3 Faculty of Physics, University of Vienna, Strudlhofgasse 4, 1090 Vienna, Austria

**Keywords:** single-walled carbon nanotube, growth, metallocene, growth kinetics, Raman spectroscopy

## Abstract

We have investigated the effects of temperature, diameter and metal catalyst type on the growth of inner nanotubes inside metallocene-filled single-walled carbon nanotubes (SWCNTs). The effects on the yield of different chiralities of inner nanotubes were scrutinized by multifrequency Raman spectroscopy. The investigated diameters range from ~0.7 to 1.3 nm and comprise 36 distinct chiralities. For all three investigated metals (Ni, Co, Fe), there is a linear correlation of growth temperature with nanotube diameter. The common slope for these metals is found to be 40.5 °C/Å. The temperature difference between the largest and the smallest diameter tubes amounts to ~230 °C for all three precursors. The growth temperatures are offset by 34 °C from Ni to Co and another 28 °C from Co to Fe. The quantified correlations of temperature, diameter and metal catalyst type provide the basis for engineering the diameter-specific growth of nanotubes.

## 1. Introduction

Single-walled carbon nanotubes (SWCNTs) possess unique chemical and physical properties, which made them an object of investigations of many researchers [1]. The synthesis methods of nanotubes in high yield and purity were developed. However, modern methods of synthesis allow obtaining mixtures of nanotubes with inhomogeneous properties due to the interconnectivity of synthesis parameters. Thus, a well-defined system for synthesis with controlled parameters is required.

In 2005, the filling of SWCNTs with metallocene molecules was firstly demonstrated [2,3]. Later on, ferrocene [4,5,6], nickelocene [7], cobaltocene [8] and cerocene [9] were encapsulated inside SWCNTs. In 2008, experiments with heating of ferrocene inside SWCNTs led to the formation of double-walled carbon nanotubes (DWCNTs) [4]. It was revealed that metal carbide or pure metal particles formed as a result of the decomposition of metallocene served as a catalyst of the inner tube growth. There is significant experience and advancement in the understanding of similarly complex nanostructured C-based compounds and the chemistry of growth using the guidance of theoretical approaches [10,11,12,13,14,15].

Metallocene-filled SWCNTs serve as a catalyst source, carbon feedstock and container, providing a shielded environment for the nanotube growth at the same time [4]. The diameter of the inner tubes can be controlled by the choice of the pristine SWCNT material. The growth properties of inner tubes inside the host SWCNTs were studied [16,17,18,19]. Qualitative dependences of the growth temperatures of inner tubes on their diameter were reported. However, a systematic investigation and quantitative study of the correlation between the growth temperature, inner tube diameter and metal catalyst type is still lacking.

**Figure 1:**
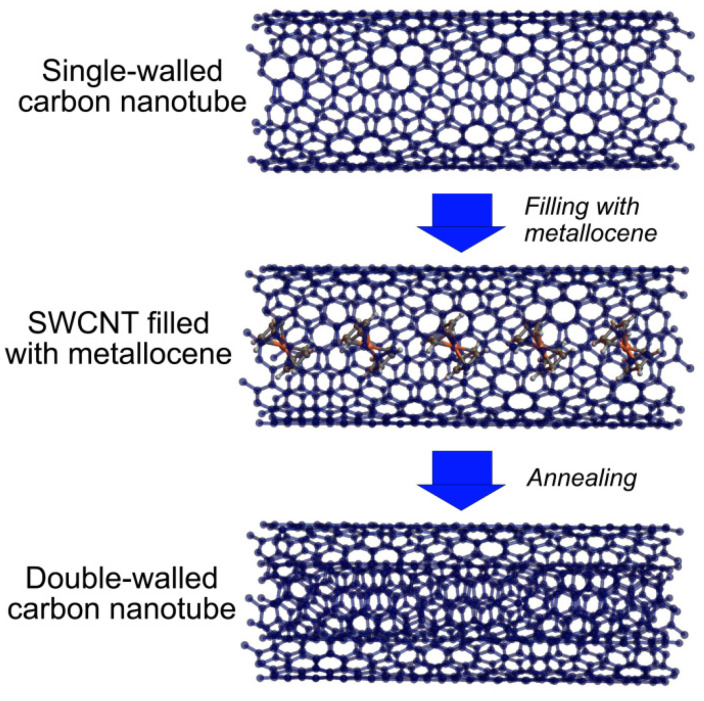
The schematics of the synthesis process of DWCNTs from metallocene-filled SWCNTs.

In this work, we close this gap by performing a systematic study of the temperature-dependent growth of 36 inner tubes with the diameters in a broad range from ~0.7 to 1.3 nm inside nickelocene- (NiCp_2_), cobaltocene- (CoCp_2_) and ferrocene (FeCp_2_)-filled SWCNTs (Figure 1). The influence of the growth temperature and type of metal catalyst on the diameter-specific growth of inner tubes is quantified. The growth temperature is found to decrease linearly with decreasing the inner tube diameter. The temperature difference between the largest and the smallest diameter tubes equals ~230 °C for all three precursors. The growth temperatures of the inner tubes inside the CoCp_2_-filled SWCNTs are higher by 34 ± 6 °C, as compared to the NiCp_2_-filled SWCNTs, and the growth temperatures of the inner tubes inside the FeCp_2_-filled SWCNTs are higher by another 28 ± 5 °C.

## 2. Materials and Methods

Nickelocene and cobaltocene were observed to decompose at temperatures higher than 60–70 °C. However, they were easily sublimated in a vacuum at temperatures as low as 50 °C, without a noticeable decomposition. Therefore, the following technique was used for the encapsulation of nickelocene and cobaltocene inside the nanotubes. The SWCNTs were annealed in air at 500 °C for 1 h to open the nanotube ends. The pre-opened SWCNT film and metallocene powder ((C_5_H_5_)_2_Ni or (C_5_H_5_)_2_Co, 99%, Strem Chemicals Inc., Bischheim, France) were placed into a glass tube (Pyrex, Chateauroux, France). This procedure was performed in a glove box in an atmosphere of argon, because metallocenes are easily oxidized in air. Then, the tube was connected to a turbopump (Pfeiffer vacuum) that provided a vacuum better than 10^−6^ mbar and evacuated for 20 min. After that, the SWCNTs and metallocene powder were sealed into an ampoule. The ampoule was heated at 50 °C for 5 days. After the filling experiment was finished, the ampoule was opened in a glove box.

Ferrocene was observed to be stable at high temperatures and in air. Therefore, another filling procedure was applied for its encapsulation into the SWCNTs. It led to a higher filling degree of the nanotubes with ferrocene than the above-described procedure. The pre-opened SWCNT film and ferrocene powder (98%, Aldrich, Darmstadt, Germany) were placed into a Pyrex-glass tube that was then connected to the turbopump, evacuated for 20 min and sealed into an ampoule. The ampoule was heated up to 350 °C, kept at this temperature for 42 h and then cooled down with the furnace to room temperature. After that, the ampoule was opened. The filled nanotube samples are labeled MCp_2_@SWCNT, where M = Ni, Co and Fe.

The annealing of the metallocene-filled nanotube samples was performed using a tube furnace (Carbolite, Neuhausen, Germany) connected to a turbopump (Pfeiffer vacuum, Vienna, Austria), providing a vacuum better than 10^−6^ mbar. The furnace was heated up to annealing temperature, which ranged between 400 and 1200 °C, kept at this temperature for 2 h and then switched off. The annealed samples were kept in a glove box under argon atmosphere.

The nanotube films obtained after annealing were studied by multifrequency Raman spectroscopy using the Horiba Jobin Yvon LabRAM HR800 spectrometer (Tulln, Austria) adapted for multifrequency measurements, as described in [20]. The system is equipped with an internal He/Ne laser operating at a wavelength of 633 nm (energy of 1.96 eV) and an external tunable Ar/Kr mixed gas laser (Coherent Innova 70c, Dieburg, Germany) operated at wavelengths of 458, 488, 514, 531, 568 and 647 nm (energies of 2.71, 2.54, 2.41, 2.34, 2.18 and 1.92 eV, respectively). For the measurements, the samples were attached to a sticky aluminum foil. The spectra were recorded in the range from 50 up to 3000 cm^−1^. A constant incident laser power of 0.5 mW, a 1000 μm pinhole, a 100 μm slit and a 600 mm^−1^ grating were used. The measurement of the complete spectral range was performed in a multiwindow regime. Every window was measured during 5 s, 12 times. One measurement lasted for about 7 min. Additionally, samples were studied using a Brucker RFS 100/S FT spectrometer (Nd:YAG laser) (Billerica, MA, USA) with a wavelength of 1064 nm (1.17 eV). A constant incident laser power ranging between 100 and 150 mW was used. Every spectrum was acquired in the range from –1400 to 3500 cm^−1^ with 4000 scans. The spectral resolution was 1 cm^−1^. The measurement time was about 6–8 h. The measurements were performed at room temperature in air.

The radial breathing mode (RBM) bands of the Raman spectra were fitted using PeakFit v4.12. For the comparison of the complete range spectra acquired at different laser wavelengths, they were normalized to the area intensity of the G-band (between 1350 and 1700 cm^−1^), in order to exclude effects of differences in focusing.

## 3. Results

### 3.1. SEM and TEM Studies

**Figure 2:**
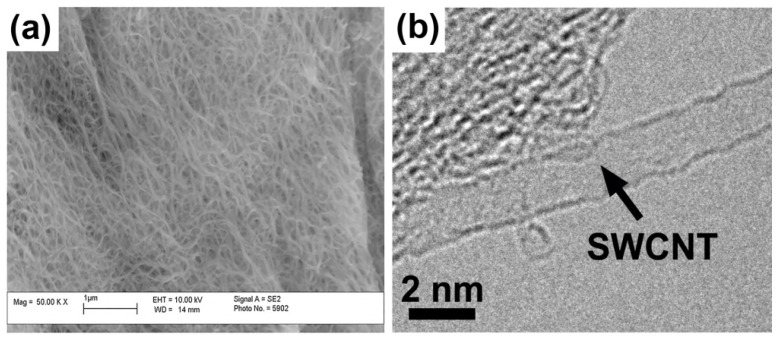
The SEM (**a**) (reprinted from [21], under the terms of the Creative Commons CC BY license) and TEM (**b**) images of the pristine SWCNTs (reprinted with permission from Springer, Applied Physics A, Kharlamova et al., Growth dynamics of inner tubes inside cobaltocene-filled single-walled carbon nanotubes) [22]. Copyright 2016).

The morphology of the pristine SWCNTs was investigated by microscopic techniques. Figure 2 shows the scanning (SEM) [21] and transmission electron microscopy (TEM) data of the pristine SWCNTs [22]. The data show that the SWCNTs represent homogenous material and have a high purity.

### 3.2. Multifrequency Raman Spectroscopy Studies on Inner Tube Growth

Multifrequency Raman spectroscopy was used to analyze the diameter distribution of inner tubes obtained from annealing NiCp_2_-, CoCp_2_- and FeCp_2_-filled SWCNTs. The use of multiple wavelengths allows combining the data from different resonance windows in the Kataura plot [23]. The peak positions of the RBM-band (*ω_RBM_*) scales with the inverse nanotube diameter (*d_t_*) were obtained as:

(1) $$\omega_{RBM}=\frac{227}{d_{t}}\sqrt{1+Cd_{t}^{2}},$$

where C = 0.05786 nm^−2^ [24]. The RBM frequencies mark all nanotube diameters, present in a macroscopic sample. We reported the Raman spectroscopy data for the pristine SWCNTs in our previous paper [25]. In RBM of SWCNTs, the peaks at frequencies between 120 and 210 cm^−1^ are observed, which correspond to diameters between ~1.2 and 2.1 nm [24]. The strongest RBM peaks are at all excitation wavelengths located between 140 and 150 cm^−1^, which is in agreement with a mean diameter of SWCNTs of ~1.7 nm. In accordance with the Kataura plot, the RBM peaks observed with laser wavelengths from 458 to 647 nm can be assigned to electronic transitions between the 3rd and 4th van Hove singularities in semiconducting SWCNTs, whereas the RBM as measured at 1064 nm is in resonance with the 2nd van Hove singularities in semiconducting SWCNTs [23]. This assignment is further backed up by the G-line showing the archetypical narrow Lorentzian profile of bulk semiconducting SWCNTs [26,27,28].

**Figure 3:**
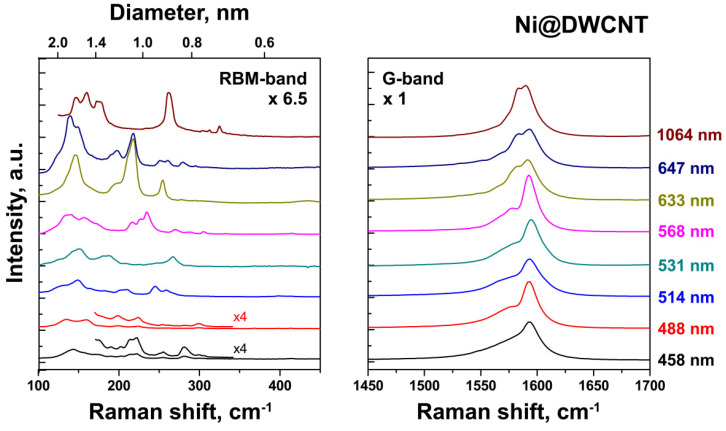
The RBM- and G-bands of the multifrequency Raman spectra of SWCNTs filled with nickelocene heated at 900 °C for 2 h.

Figure 3 demonstrates the RBM- and G-band regions of multifrequency Raman spectra of NiCp_2_-filled SWCNTs annealed at 900 °C for 2 h. The spectra of DWCNTs obtained from SWCNTs filled with NiCp_2_, CoCp_2_ and FeCp_2_ are very similar. They show additional peaks of inner tubes between 170 and 330 cm^−1^, which correspond to diameters between ~0.7 and 1.3 nm, respectively [24]. In contrast to the pristine SWCNTs, there are also metallic inner tubes in resonance. The small-diameter inner tubes also give rise to an additional 10 cm^−1^ downshifted component in the G-line.

### 3.3. Evaluation of Growth Temperatures of Inner Tubes

To evaluate growth temperatures of inner tubes, metallocene-filled SWCNTs were heated at temperatures between 385 and 1000 °C for 2 h. The NiCp_2_-filled SWCNTs were annealed at temperatures between 385 and 1000 °C, and CoCp_2_- and FeCp_2_-filled SWCNTs were annealed at temperatures between 500 and 1000 °C. In these temperature ranges, the growth of inner tubes occurred. The minimal temperature was chosen as the temperature at which the growth of inner tubes starts. The maximal temperature was chosen as the temperature at which the growth of inner tubes is completed.

**Figure 4:**
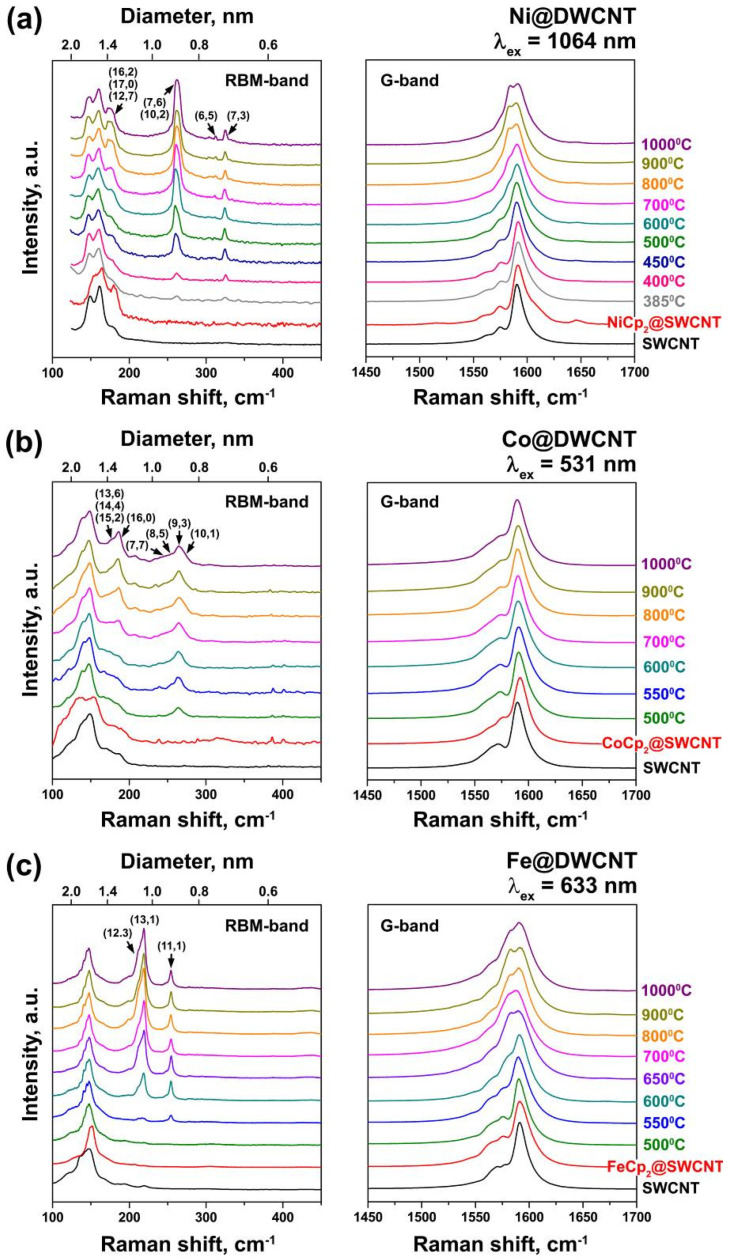
The RBM- and G-bands of Raman spectra of (**a**) the empty SWCNTs, the nanotubes filled with nickelocene and the filled SWCNTs heated from 385 to 1000 °C for 2 h acquired with the 1064 nm laser (E_ex_ = 1.17 eV), (**b**) the empty SWCNTs, the nanotubes filled with cobaltocene and the filled SWCNTs heated from 500 to 1000 °C for 2 h acquired with the 531 nm laser (E_ex_ = 2.34 eV) (reprinted with permission from Springer, Applied Physics A, Kharlamova et al., Growth dynamics of inner tubes inside cobaltocene-filled single-walled carbon nanotubes) [22]. Copyright 2016) and (**c**) the empty SWCNTs, the nanotubes filled with ferrocene and the filled SWCNTs heated from 500 to 1000 °C for 2 h acquired with the 633 nm laser (E_ex_ = 1.96 eV). The chiral indexes of inner tubes are indicated.

The Raman spectra of the annealed samples were investigated. The changes in the Raman spectra were used to quantify the relative yield of different diameter inner nanotubes for NiCp_2_-, CoCp_2_- and FeCp_2_-filling. Figure 4 shows selected temperature series for the different metallocenes at different wavelengths. As the very same inner nanotubes meet the resonance conditions at a given wavelength irrespective of the used metallocenes, the different metallocenes only differ in relative intensities of RBM peaks for their different diameter-dependent yield of inner nanotubes. The RBM peaks of inner nanotubes are identified according to the Kataura plot [23] and labeled in Figure 4. They show an analogous dependence on annealing temperature. There is an onset temperature where the RBM starts appearing, and then it increases until a saturation temperature. The highest temperature of 1000 °C guarantees completed saturation in all cases. The saturated inner tube RBM intensities match those of the outer nanotubes, and testify to a uniform high-yield filling for all three different metallocenes. The growing RBM peaks of inner nanotubes are concomitant with an additional signature in the G-band, which is attributed to the downshifted components of the inner nanotubes. The data in Figure 4 also suggest that there is a strong influence of inner tube diameter as well as a dependence on the metal or metallocene type on the onset and saturation temperature of the inner tube growth.

For instance, with Ni in Figure 4a after heating at 385 °C, the RBM peaks of the (7,6), (10,2), (6,5) and (7,3) tubes are observed. The peaks of the (16,2), (17,0) and (12,7) tubes with the largest diameters only appear after annealing at 600 °C. The saturated intensity of the peaks of the (6,5) and (7,3) tubes is reached at 450 °C, the (7,6) and (10,2) tubes—at 600 °C and the (16,2), (17,0) and (12,7) tubes—at 900 °C. With Co in Figure 4b, the joint RBM of the (7,7), (8,5), (9,3) and (10,1) tubes is large after heating at 500 °C. Its intensity saturates at 700 °C. The (13,6), (14,4), (15,2) and (16,0) tubes with greater diameters start to be observed at a temperature of 600 °C, and their intensities saturate at 900 °C. With Fe in Figure 4c, the RBM peak of the smallest diameter (11,1) tube only appeared in the spectrum after annealing at 550 °C, and saturated at 600 °C. The peaks of the (12,3) and (13,1) tubes have noticeable intensity after annealing at 600 °C and saturate at 800 °C.

**Figure 5:**
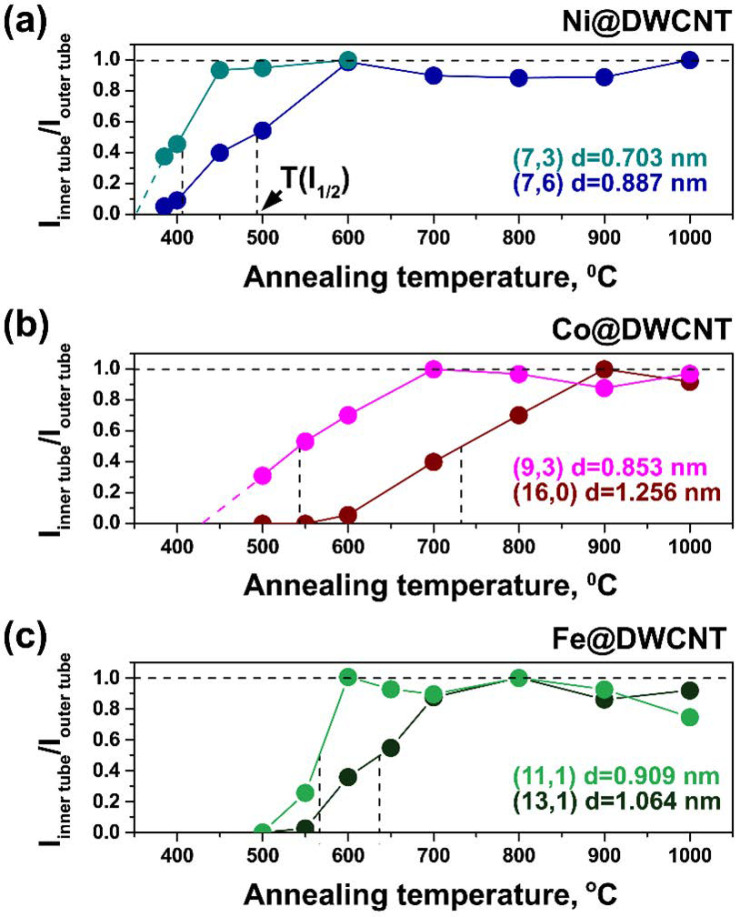
The normalized intensities ratio, I_inner tube_/I_outer tube_, plotted against annealing temperature for (**a**) the (7,6) and (7,3) tubes formed inside the heated nickelocene-filled SWCNTs, (**b**) the (16,0) and (9,3) tubes formed inside the heated cobaltocene-filled SWCNTs and (**c**) the (13,1) and (11,1) tubes formed inside the heated ferrocene-filled SWCNTs. T(I_1__/__2_) is marked by dashed vertical lines.

Figure 5 presents the selected dependences of the intensity of the inner tube RBM peak normalized to the outer tube peak intensity for the most intense RBM peaks visible in Figure 4. These are the (7,6) and (7,3) tubes obtained from NiCp_2_-filled SWCNTs (Figure 4a or Figure 5a), the (16,0) and (9,3) tubes obtained from CoCp_2_-filled SWCNTs (Figure 4b or Figure 5b) and the (13,1) and (11,1) tubes obtained from FeCp_2_-filled SWCNTs (Figure 4c or Figure 5c). In all cases, there is an onset temperature after which the RBM is visible and a saturation temperature after which the intensity is constant. In all three panels, these temperatures are notably lower for the smaller diameter inner nanotubes.

The temperature, T(I_1/2_), at which the intensity of the inner tube RBM peak reaches half of its maximum after 2 h of annealing time was determined, as denoted by dashed vertical lines in Figure 5. The T(I_1/2_) temperature equals 490 and 405 °C for the (7,6) and (7,3) inner tubes, 730 and 540 °C for the (16,0) and (9,3) tubes and 635 and 565 °C for the (13,1) and (11,1) tubes, respectively. The obtained plots in Figure 5 show a diameter-dependence of T(I_1/2_): the growth temperature becomes larger with increasing the tube diameter. Moreover, the T(I_1/2_) temperatures reveal a dependence on the metal type in the metallocene precursor. For the (7,6), (9,3) and (11,1) inner tubes with similar diameters that are grown from the NiCp_2_-, CoCp_2_- and FeCp_2_-filled SWCNTs respectively, the T(I_1/2_) growth temperature increases gradually. This points to an increase of T(I_1/2_) temperatures of inner tubes in line with Ni-Co-Fe.

### 3.4. Dependence of Growth Temperatures of Inner Tubes on Their Diameter and Metal Type

**Figure 6:**
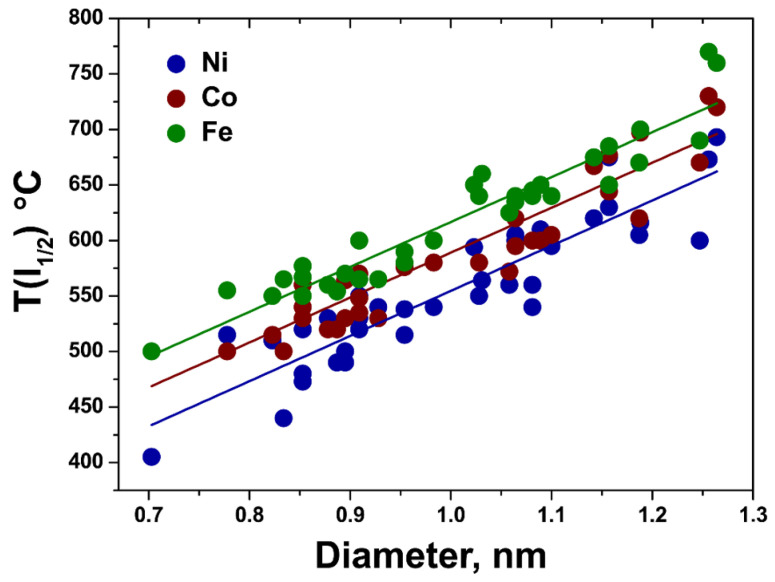
T(I_1/2_) plotted against the tube diameter for the tubes grown inside the annealed NiCp_2_-, CoCp_2_- and FeCp_2_-filled SWCNTs, observed in the Raman spectra acquired at laser wavelengths between 458 and 1064 nm. The linear fits of the experimental data (filled circles) are shown as solid lines.

To investigate the full diameter- and metal type-dependence of the growth temperature of inner tubes, the T(I_1/2_) temperatures were evaluated for the inner tubes observed in the Raman spectra of the annealed NiCp_2_-, CoCp_2_- and FeCp_2_-filled SWCNTs acquired at all 8 laser wavelengths (Figure 3). Table 1 summarizes the detected inner tubes in the order of increasing diameter and also T(I_1/2_) growth temperatures. For the tubes with the diameter ranging from 0.703 to 1.264 nm, the T(I_1/2_) temperature varies between 405 °C for Ni and 770 °C for Fe. Figure 6 shows the plot of the T(I_1/2_) growth temperature versus the tube diameter for the tubes formed inside the NiCp_2_-, CoCp_2_- and FeCp_2_-filled SWCNTs. It demonstrates the linear increase of the growth temperature with increasing tube diameter. The slopes are 40.7 ± 3.2, 40.5 ± 3.1 and 40.4 ± 2.2 °C/Å for Ni, Co and Fe, respectively. The linear fitting of the data reveals that the growth temperature of the inner tubes inside the CoCp_2_-filled SWCNTs is higher by 34 ± 6 °C as compared to the NiCp_2_-filled SWCNTs, and that the growth temperature inside the FeCp_2_-filled SWCNTs is higher by another 28 ± 5 °C. The temperature difference between the precursors is constant within the considered diameter range of the inner tubes.

## 4. Discussion

The annealing of metallocene-filled SWCNTs leads to the decomposition of molecules with the formation of metal carbides and pure metals [7,21,29,30]. The encapsulated metal carbides and metals serve as catalysts for the inner tube growth. Thus, the growth mechanism implies the stages of (i) decomposition of metallocene and (ii) growth of inner nanotubes on the metal carbide and metal particles. In [21,29,30], it was revealed that the growth mechanism of inner tubes includes two successive stages of the growth on carburized and purely metallic catalytic particles. Each stage is characterized with different growth rates and activation energies.

The obtained data reveal that the growth rates of the largest diameter tubes (*d*_t_~1.3 nm) are the same as the ones of the smallest diameter tubes (d_t_~0.7 nm) formed from the same metallocene precursor if the growth temperature is increased by ~230 °C. This is caused by increased catalytic activity of smaller diameter metallic particles [31,32]. Similar trends were previously observed for ferrocene [4,12], nickelocene [13] and Pt (II) acetylacetonate-filled SWCNTs [33,34], and for the CVD growth of carbon filaments and nanotubes [35,36,37,38,39,40].

The revealed offset of the growth temperature between nickelocene, cobaltocene and ferrocene demonstrates that the growth rates of the same inner tubes with the cobaltocene precursor are the same as the ones with the nickelocene precursor if the growth temperature is increased by 34 ± 6 °C, and are also the same on Fe if the temperature is raised by another 28 ± 5 °C. This temperature equivalence is in agreement with the reported diffusion rates of carbon in metals [31,41]. If the growth temperature is sufficiently high, the catalytic activity of smaller and bigger catalyst particles is high enough. The growth is then limited by the rate at which carbon can be supplied to the catalyst particles. Unlike the catalytic activity, the feed rate does not depend on the diameter.

We would reason that the growth rate is determined by the growth rate-limiting process. This is at lower temperatures the catalytic activity and at higher temperatures the diffusion rate of carbon. The rates should scale inversely with the T(I_1/2_) growth time. Therefore, in the regime where the catalytic activity is the limiting process, the growth rate scales inversely with the radius.

It should be noted that significantly larger growth temperatures were reported for cerocene [9] and Pt (II) acetylacetonate [33,34] precursors. Decreasing the growth temperature of the inner tube opens the possibility to synthesize nanotubes under ambient conditions. The revealed correlations also show that the growth rates are equivalent for 1.6 Å larger diameter nanotubes when switching from Fe to Ni. The obtained linear dependences provide the proper scaling for equivalent changes in diameter, temperature and metal type on the growth rates of nanotubes. If one of the three parameters is changed, the required compensations in either of the other two are known.

The obtained nanostructures have unique electronic properties [5,7,9,18,19]. The filling of SWCNTs with metallocene molecules opens the way of *n*-doping of the nanotubes, and the further annealing of the filled SWCNTs allows modifying the electronic properties of SWCNTs in a tailored manner. Combining the controlled growth kinetics of inner SWCNTs [21,29,30] and tailored electronic properties of these nanostructures [42,43] allows applying these systems in various fields, such as nanoelectronics, thermoelectric power generation, catalysis, sensors, electrochemical energy storage, spintronics, magnetic recording and biomedicine [44,45,46,47].

## 5. Conclusions

To summarize, a systematic study of the temperature-dependent growth of 36 inner tubes with the diameters in a broad range from ~0.7 to 1.3 nm inside the nickelocene-, cobaltocene- and ferrocene-filled SWCNTs was conducted. The growth temperatures, T(I_1/2_), at which the intensity of the inner tube RBM peak in the Raman spectra reaches half of its maximum were evaluated. The influence of the growth temperature and type of metal catalyst on the diameter-specific growth of inner tubes was quantified. The growth temperature was found to decrease linearly with decreasing the inner tube diameter. This means that the smallest nanotubes grow the fastest. The temperature difference between the largest and the smallest diameter tubes amounted to ~230 °C for all three precursors. The growth temperatures increase in line with nickelocene-cobaltocene-ferrocene. The growth temperatures of the inner tubes inside the CoCp_2_-filled SWCNTs were higher by 34 ± 6 °C, as compared to the NiCp_2_-filled SWCNTs, and the growth temperatures of the inner tubes inside the FeCp_2_-filled SWCNTs were higher by another 28 ± 5 °C. Therefore, for controlling the diameter of inner tubes, one should choose an appropriate precursor and, more importantly, the growth temperature. These findings provide the basis for tailoring the diameter-specific growth of nanotubes.

## Figures and Tables

**Table 1:** The diameters (*d*_t_), chiral indexes (n, m), RBM peak positions, excitation laser wavelengths (λ_ex_) and evaluated T(I_1/2_) growth temperatures of the inner tubes observed in the Raman spectra of the annealed NiCp_2_-, CoCp_2_- and FeCp_2_-filled SWCNTs.

*d*_t_, nm	(n,m)	RBM, cm^−1^	λ_ex_, nm	T(I_1/2_), °C		
				Precursor		
				NiCp_2_	CoCp_2_	FeCp_2_
0.703	(7,3)	325	1064	405	500	500
0.778	(8,3)	295	647	515	500	555
0.823	(7,5)	279	647	510	515	550
0.834	(8,4)	279	458	440	500	565
0.853	(9,3)	267	514	480	530	567
0.853	(9,3)	265	531	473	540	577
0.853	(9,3)	269	568	520	560	550
0.878	(10,2)	264	1064	530	520	560
0.887	(7,6)	260	1064	490	520	554
0.895	(8,5)	258	514	500	564	570
0.895	(8,5)	255	531	490	530	570
0.909	(11,1)	253	458	530	548	600
0.909	(11,1)	254	633	520	535	565
0.909	(11,1)	256	647	550	570	565
0.928	(10,3)	251	647	540	530	565
0.954	(7,7)	245	514	515	590	590
0.954	(7,7)	247	531	538	576	580
0.983	(10,4)	235	568	540	580	600
1.023	(13,0)	230	488	594	650	650
1.028	(9,6)	226	568	550	580	640
1.031	(12,2)	225	488	564	660	660
1.058	(11,4)	221	458	560	572	625
1.064	(13,1)	219	633	600	595	635
1.064	(13,1)	219	647	605	620	640
1.081	(12,3)	214	633	540	600	640
1.081	(12,3)	216	647	560	600	645
1.089	(8,8)	215	568	610	600	650
1.100	(10,6)	212	458	595	605	640
1.142	(14,1)	207	514	620	667	675
1.157	(9,8)	202	458	675	677	650
1.157	(13,3)	202	514	630	644	685
1.187	(14,2)	196	647	605	620	670
1.188	(12,5)	200	488	616	697	700
1.247	(12,6)	190	647	600	670	690
1.256	(16,0)	186	531	673	730	770
1.264	(15,2)	184	531	693	720	760

## Data Availability

The data are available on request from authors.
